# Anti-Inflammatory and Antihyperalgesic Activities of Ethanolic Extract and Fruticulin A from *Salvia lachnostachys* Leaves in Mice

**DOI:** 10.1155/2014/835914

**Published:** 2014-11-10

**Authors:** Ana Claudia Piccinelli, Diana Figueiredo de Santana Aquino, Priscila Neder Morato, Ângela Midori Kuraoka-Oliveira, Regiane Lauriano Batista Strapasson, Élide Pereira dos Santos, Maria Élida Alves Stefanello, Rodrigo Juliano Oliveira, Cândida Aparecida Leite Kassuya

**Affiliations:** ^1^School of Health Sciences, Federal University of Grande Dourados (UFGD), Rodovia Dourados, Itahum, Km 12, Cidade Universitária, Caixa Postal 533, 79.804-970 Dourados, MS, Brazil; ^2^School of Biological and Health Sciences, University Center of Grande Dourados, 79.824-900 Dourados, MS, Brazil; ^3^Chemistry Department, Federal University of Paraná, 81531-980 Curitiba, PR, Brazil; ^4^Botany Department, Federal University of Paraná, 81531-980 Curitiba, PR, Brazil; ^5^“Dr. Hélio Mandetta” School of Medicine, Federal University of Mato Grosso do Sul, 79070-900 Campo Grande, MS, Brazil

## Abstract

The anti-inflammatory and analgesic effects of the ethanolic extract (SLEE) and fruticulin A from the leaves of *Salvia lachnostachys* were evaluated in mice, using experimental models of inflammation (paw oedema and pleurisy induced by carrageenan injection) and hyperalgesia (electronic Von Frey). Oral administration of SLEE (30, 100, and 300 mg/kg) and fruticulin A (0.3 and 3.0 mg/kg) decreased the total leucocytes number in pleural lavage, protein extravasation, and paw oedema. SLEE (100 mg/kg) and fruticulin A (3 mg/kg) also exhibited antihyperalgesic activity in carrageenan induced mechanical hyperalgesia. In addition, fruticulin A (3 mg/kg) prevented mechanical hyperalgesia, inhibiting TNF but not L-DOPA-induced mechanical hyperalgesia. In conclusion, SLEE and fruticulin A display anti-inflammatory and analgesic properties. Therefore, fruticulin A is at least partially responsible for the activity observed in the ethanolic extract of *Salvia lachnostachys*.

## 1. Introduction

The current therapy for inflammation and pain frequently causes undesirable side effects, especially in the management of chronic diseases, since the patients must take the remedies for long time spans. Thus, studies for the discovery of safer and more efficient drugs are required. Plants produce a great diversity of secondary metabolites, being a natural source of bioactive compounds [1, 2].

The genus* Salvia* (Lamiaceae) comprises around 900 species distributed in tropical and subtropical regions. Several species have been used in the traditional medicine for treating diverse ailments such as aches, epilepsy, colds, bronchitis, tuberculosis, hemorrhage, and menstrual disorders. Pharmacological studies have confirmed the importance of* Salvia* species as sources of antimicrobial, antioxidant, and anti-inflammatory compounds [3]. In Brazil, the genus is represented by 68 species of shrubs, subshrubs, and herbs distributed in the Southern, South-Eastern, and Mid-Western regions of the country [4].

**Figure 1:**
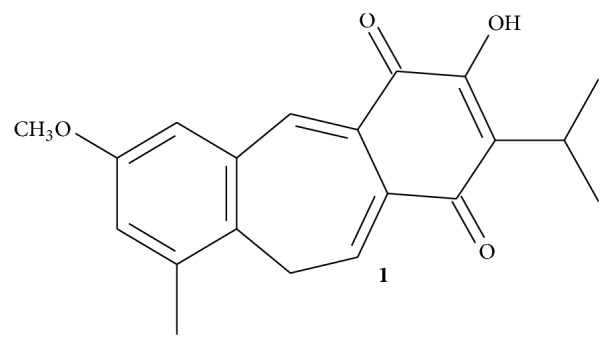
Structure of fruticulin A.

*S. lachnostachys *Benth. is an herbaceous plant, slightly aromatic, endemic to southern Brazil. Previous chemical studies have reported the chemical composition of its essential oils [5], the isolation of the triterpenes ursolic and oleanolic acids, and the diterpene fruticulin A (Figure 1) from the ethanolic extract of leaves [6]. To our knowledge, this plant is not used in traditional medicine in Brazil.

The lack of pharmacological studies with* S. lachnostachys* led us to investigate the anti-inflammatory and antihyperalgesic effects of the ethanolic extract of* Salvia lachnostachys* leaves and that of fruticulin A, one major compound of the extract, in experimental models of inflammation and hyperalgesia in mice.

## 2. Materials and Methods

### 2.1. Plant Material

Leaves were collected from natural populations in Curitiba, Paraná state, Brazil (25°30′44.6′′S, 49°18′7.13′′W), and a voucher was deposited in the Herbarium of the Federal University of Paraná (UPCB 1251).

### 2.2. Preparation of the Ethanolic Extract and Isolation of Fruticulin A

Dried and powdered leaves of* S. lachnostachys* were extracted with hexane followed by ethanol, yielding the ethanolic extract (SLEE, 3.7%) after solvent removal. Fruticulin A (**1**) was isolated by chromatographic fractionation of SLEE, yielding around 1%. Detailed experimental procedures were previously described [6].

### 2.3. Animals

The experiments were made using adult male and female* Swiss* mice (20–30 g, 50 days old), housed under a 12 h light/dark cycle, with controlled temperature (23 ± 1°C) and water and food* ad libitum*. All experimental procedures were approved by the ethics committee of UFGD for animal uses (Nbr. 013/2013).

### 2.4. Reagents

*λ*-Carrageenan (Cg), TNF, L-DOPA, Bradford reagent, and dexamethasone (DEXA) were purchased from Sigma-Aldrich Co. LLC (St. Louis, MO, USA).

### 2.5. Carrageenan-Induced Paw Oedema

SLEE and** 1** were tested in three doses, which were determined in a pilot study and according to literature data [7]. Male animals were divided into experimental groups (*n* = 5 animals/group). Group 1 (negative control group) was treated orally with vehicle (10 mL/kg of saline 0.9%). Different groups of mice received SLEE (30, 100, and 300 mg/kg) or** 1** (0.3, 1.0, and 3.0 mg/kg) dissolved in 0.9% of saline solution by oral route. The positive control group received DEXA (1.0 mg/kg) by subcutaneous route. After 1 h, the animals received an injection of Cg (300 *μ*g/paw, 50 *μ*L in sterile 0.9% saline) in the right paw. The contralateral paw received only saline and was used as control. Oedema was measured after 0.5, 1, 2, and 4 h with a paw plethysmometer (PANLAB Harvard) [8].

### 2.6. Carrageenan-Induced Pleurisy

Female animals were divided into experimental groups (*n* = 5 animals/group). Control that received vehicle (negative control group), SLEE (30, 100, and 300 mg/kg), and** 1** (0.3, 1.0, and 3.0 mg/kg) were given orally 1 h before Cg injection. Positive control group received 1.0 mg/kg of DEXA, subcutaneously, 1 h before Cg application. Pleurisy was induced applying Cg (300 *μ*g, 0.25 mL in phosphate buffered saline PBS, pH = 7.4) in the mice pleural cavity [9]. After 4 h, the animals were euthanized and the pleural inflammatory exudate was collected through pleural lavage with 1.0 mL of sterile saline. The exudate volume was measured, and an aliquot of 50 *μ*L was diluted in Turk's solution (1 : 20). Total leukocytes were counted in a Neubauer chamber, considering the four external quadrants, with a light microscope. Protein concentration in the exudates was determined by the Bradford method.

### 2.7. Carrageenan-Induced Hyperalgesia

Mice were housed in containment boxes (W × D × H 230 × 200 × 180 mm, Insight) in a steel mesh with 1 cm diameter spacing for 1 hour, and the digital analgesymeter (Insight, EFF 301, digital analgesymeter, Von Frey) was used to determine the similar mean baseline of mechanical stimulus in the right hind paw [10, 11]. At the following day, each group of male mice (*n* = 6/group) orally received (gavage) 0.9% saline solution (negative control group), SLEE (300 mg/kg), or** 1** (3 mg/kg). After 1 h, the animals received Cg injection (300 *μ*g) subcutaneously in the right hind paw. Then, each animal was housed in the same containment boxes under the same steel mesh, and mechanical hyperalgesia was measured again after 3 and 4 h. The same procedure was performed with an injection of** 1** (1 *μ*g/paw, 20 *μ*L) and a control group (saline 0.9% v/v) to evaluate local antihyperalgesic activity of the compound directly into the paw [12].

### 2.8. TNF and L-DOPA-Induced Hyperalgesia

In order to studythe effect of** 1** on hyperalgesia induced by different* stimuli*, basal mechanical withdrawal threshold of mice was assessed with a digital analgesymeter as described above, and then the animals were treated with** 1** (1 *μ*g/paw, 20 *μ*L) or the same volume of vehicle (saline 0.9% v/v). After 15 min, they received TNF (1 pg/paw, in 20 *μ*L) or L-DOPA (10 *μ*g/paw, in 20 *μ*L) [13, 14]. Mechanical hyperalgesia was measured after 3 h [12].

### 2.9. Statistical Analysis

Data are presented as mean ± S.E.M. Differences between groups were evaluated by analysis of variance (one-way ANOVA) followed by the Newman-Keuls test or Bonferroni test. Statistical differences were considered to be significant with *P* < 0.05.

## 3. Results

### 3.1. Effects of SLEE and **1** against Carrageenan-Induced Oedema

**Figure 2:**
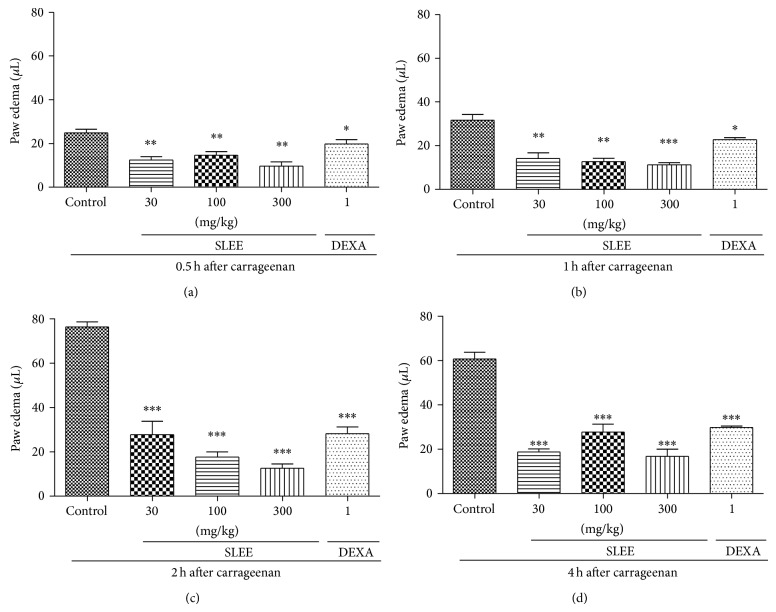
Effect of oral administration of SLEE on carrageenan-induced paw oedema in mice. Animals received SLEE (30, 100, or 300 mg/kg, p.o.) or vehicle or dexamethasone (DEXA, 1 mg/kg, s.c.) and after 1 h an intraplantar injection of carrageenan (300 *μ*g/paw). Graphics (a), (b), (c), and (d) represent the evaluation of paw oedema after 0.5, 1, 2, and 4 h, respectively, after carrageenan injection. Each bar represents the mean ± SEM of 5 animals. ^*^ *P* < 0.05, ^**^ *P* < 0.01 when compared with the control-treated group. Differences between groups were analyzed by analysis of variance (one-way ANOVA) followed by the Newman-Keuls test.

Oral treatment with SLEE significantly inhibited oedema formation in a dose-dependent manner. The maximum inhibitions were 84 ± 3% at the dose of 300 mg/kg and 77 ± 3% at the dose of 100 mg/kg after 2 hours of Cg injection, with *P* < 0.01 (Figure 2).

**Figure 3:**
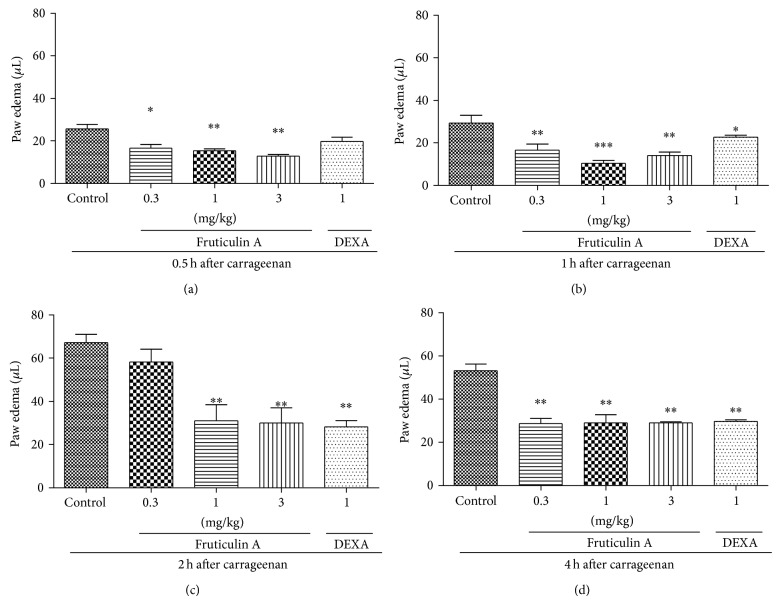
Effect of oral administration of fruticulin A on carrageenan-induced paw oedema in mice. Animals received fruticulin A (0.3, 1, and 3 mg/kg, p.o.) or vehicle or dexamethasone (DEXA, 1 mg/kg, s.c.) and after 1 h an intraplantar injection of carrageenan (300 *μ*g/paw). Graphics (a), (b), (c), and (d) represent the evaluation of paw oedema after 0.5, 1, 2, and 4 h, respectively, after carrageenan injection. Each bar represents the mean ± SEM of 5 animals. ^*^ *P* < 0.05, ^**^ *P* < 0.01 when compared with the control-treated group. Differences between groups were analyzed by analysis of variance (one-way ANOVA) followed by the Newman-Keuls test.

Statistical analysis with ANOVA and posttest Bonferroni showed that all doses tested of SLEE (except the group treated with 30 mg/kg, Figure 2(c)), fruticulin A, and DEXA (Figures 2 and 3) were statistically different from the control group. The tested doses of SLEE were not statistically different among themselves or to DEXA, except for SLEE 300 mg/kg after 2 h of oedema induction (Figure 2(c)).

We also observed that the administration of** 1** significantly decreased paw oedema in mice at doses of 1.0 and 3.0 mg/kg (Figure 3(c)), with no significant difference between the doses. The maximum inhibitions were 54 ± 11% at the dose of 1 mg/kg and 55 ± 11% at the dose of 3 mg/kg after 2 hours of Cg injection, with *P* < 0.05. The tested doses were not statistically different among themselves or to DEXA, except for the dose of 30 mg/kg after 2 h of oedema induction (Figure 3).

### 3.2. Anti-Inflammatory Activity of SLEE and **1** on Carrageenan-Induced Leukocyte Migration and Protein Extravasation in Pleural Cavity

**Figure 4:**
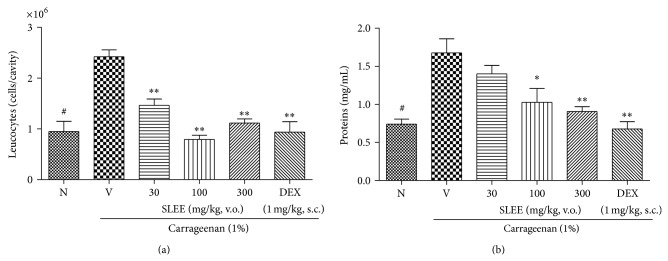
Effect of oral administration of SLEE at inhibition of the leukocyte migration (a) and protein extravasation (b) on pleurisy test in mice. Mice were treated one hour before an intrapleural injection of carrageenan, with SLEE (30, 100, and 300 mg/kg p.o.), dexamethasone (DEXA, 1 mg/kg, s.c.), or saline solution (V). Naive group (N), also treated with saline p.o., received an intrapleural injection of sterile saline. Pleural cavity was washed with PBS/EDTA 10 mM. Cells were counted and plasma leakage was analyzed. In (a), number of cells that migrated to pleural cavity 4 hours after carrageenan injection. In (b), plasma leakage measured by Bradford's reaction. The bars express the mean ± SEM of 5 animals. The symbol ∗ compared vehicle (V) versus SLEE or DEX treated groups and # compared the vehicle treated group with naive. ^#^ *P* < 0.001; ^*^ *P* < 0.05; ^**^ *P* < 0.01. Differences between groups were analyzed by analysis of variance (one-way ANOVA) followed by the Newman-Keuls test.

In the pleurisy test, the number of total leukocytes counted in a Neubauer chamber decreased significantly (*P* < 0.01) for mice treated with 30, 100, or 300 mg/kg of SLEE (Figure 4(a)) when compared to the control group, with no significant difference between the doses. The maximum inhibitions were, respectively, 46 ± 6% and 24 ± 6%.

**Figure 5:**
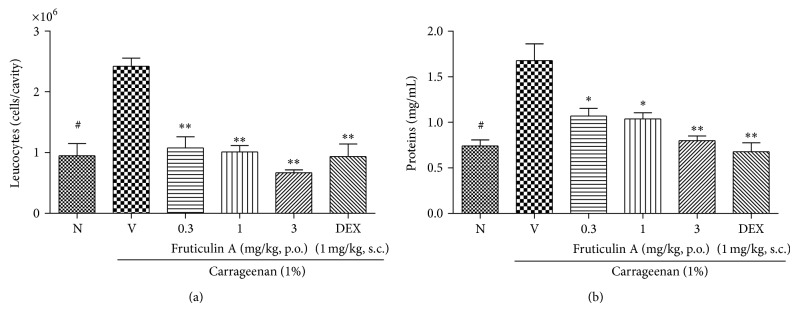
Effect of oral administration of fruticulin A at inhibition of the leukocyte migration (a) and protein extravasation (b) on pleurisy test in mice. Mice were treated one hour before an intrapleural injection of carrageenan, with fruticulin A (0.3, 1, and 3 mg/kg p.o.), dexamethasone (DEXA, 1 mg/kg, s.c.), or saline solution (V). Naive group (N), also treated with saline p.o., received an intrapleural injection of sterile saline. Pleural cavity was washed with PBS/EDTA 10 mM. Cells were counted and plasma leakage was analyzed. In (a), number of cells that migrated to pleural cavity 4 hours after carrageenan injection. In (b), plasma leakage measured by Bradford's reaction. The symbol ∗ compared vehicle (V) versus fruticulin A or DEXA treated groups and # compared the vehicle and treated group with naive. ^#^ *P* < 0.001; ^*^ *P* < 0.05; ^**^ *P* < 0.01. Differences between groups were analyzed by analysis of variance (one-way ANOVA) followed by the Newman-Keuls test.

There was also a significant decrease in pleural lavage protein concentration for groups treated with 100 and 300 mg/kg of SLEE (*P* < 0.01) when compared to the control group (Figure 4(b)). The positive control showed decreased leukocyte migration and protein extravasation (Figure 4). Compound** 1** showed significant effects (*P* < 0.01) in all tested concentrations, decreasing the total leukocyte count in a dose-dependent manner when compared to the control group (Figure 5(a)). The maximum inhibitions were, respectively, 31 ± 7% and 54 ± 3%.

The amount of protein measured in pleural lavage was also significantly lower for groups treated with** 1** when compared to the control group, with *P* < 0.05 at the dose of 0.3 and 1 mg/kg and *P* < 0.01 at dose of 3 mg/kg (Figure 5(b)).

Analyzing the results by ANOVA with posttest Bonferroni, naive group differed significantly from negative control group in Figures 4 and 5. Moreover, all doses of SLEE tested (except the group treated with 30 mg/kg, Figure 4(b)),** 1**, and DEXA (Figures 2 and 3) had statistical differences from negative control group. In Figure 4(a), the group treated with SLEE at dose of 100 mg/kg differed from other groups. And in Figure 5(a), the group treated with DEXA had statistical differences from other groups. All doses tested of SLEE did not differ between groups and dexamethasone group in Figure 5.

### 3.3. The Antihyperalgesic Activity of Oral Administration of SLEE and Local and Oral Administration of **1** on Carrageenan-Induced Mechanical Sensitivity in Mice

**Figure 6:**
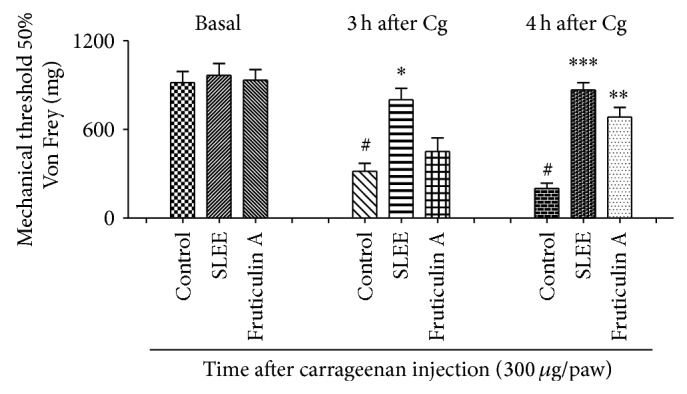
Effect of oral administration of SLEE or fruticulin A at paw withdraw threshold, on Von Frey test in mice. Animals received SLEE (300 mg/kg) or fruticulin A (3 mg/kg) or vehicle and after one hour 300 *μ*g of carrageenan injection in the right hind paw. Mechanical hyperalgesia was measured at the times 3 and 4 hours after carrageenan injection using the digital analgesymeter. The bars express the mean ± SEM of 6 animals. The symbol ∗ compared vehicle (V) versus SLEE or fruticulin A treated groups and # compared the control group before (basal) and after carrageenan treatment. ^#^ *P* < 0.001; ^*^ *P* < 0.05; ^**^ *P* < 0.01;  ^***^ *P* < 0.001. Differences between groups were analyzed by analysis of variance (one-way ANOVA) followed by the Newman-Keuls test.

Carrageenan injection decreased significantly the basal values, measured by electronic Von Frey mechanical stimulation. It is possible to see in Figure 6 that the substances orally tested relieved hyperalgesia so that the animals withstood the strength applied with the Von Frey filaments. Maximum inhibitions were 61 ± 15% for SLEE (300 mg/kg) after 3 hours of Cg injection and 72 ± 13% and 53 ± 11% for SLEE (300 mg/kg) and** 1** (3 mg/kg), respectively, after 4 hours of Cg injection.

**Figure 7:**
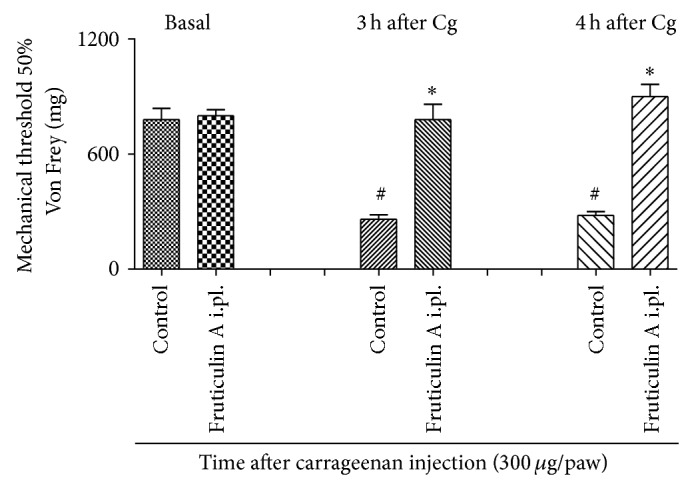
Effect of local administration of fruticulin A at paw withdraw threshold, on Von Frey test in mice. Animals received fruticulin A (1 *μ*g/paw) or vehicle and after one hour 300 *μ*g of carrageenan injection in the right hind paw. Mechanical hyperalgesia was measured at the times 3 and 4 hours after carrageenan injection using the digital analgesymeter. The bars express the mean ± SEM of 6 animals. The symbol ∗ compared vehicle (V) versus fruticulin A treated groups and # compared the control group before basal and after carrageenan treatment. ^#^ *P* < 0.001; ^*^ *P* < 0.05. Differences between groups were analyzed by analysis of variance (one-way ANOVA) followed by the Newman-Keuls test.

Local administration of compound** 1** (1 *μ*g/paw, 20 *μ*L) significantly prevented the hyperalgesia induced by carrageenan showing that compound** 1**, and not a metabolite, directly inhibits the sensibilization, with maximum inhibition of 72 ± 15% after 3 hours and 72 ± 15% after 4 hours of Cg injection, with *P* < 0.05 (Figure 7).

### 3.4. The Antiallodynic Activity of Local Administration of Fruticulin A on TNF or L-DOPA-Induced Hypernociception

**Figure 8:**
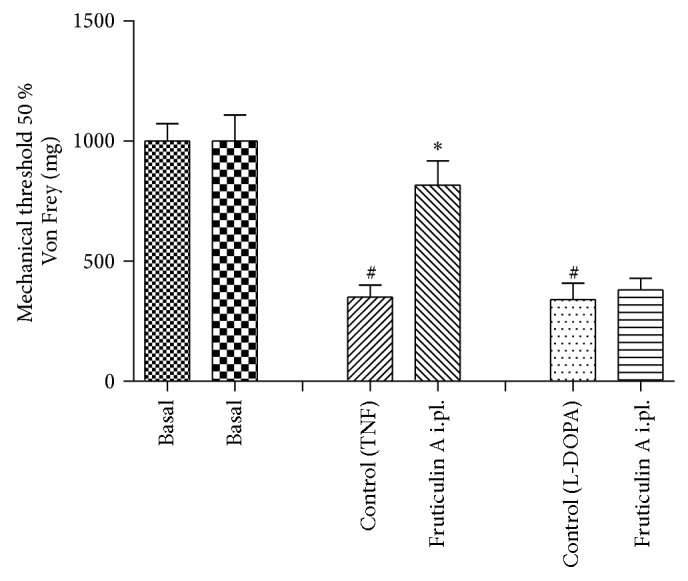
Effect of local administration of fruticulin A at paw withdraw threshold, on mechanical allodynia induced by TNF or L-DOPA in mice. Animals received fruticulin A (1 *μ*/paw) or vehicle and after 15 minutes 1 pg/paw of TNF or L-DOPA injection in the right hind paw. Mechanical hyperalgesia was measured at the times 3 hours after TNF or L-DOPA injection using the digital analgesymeter. The bars express the mean ± SEM of 6 animals. The symbol ∗ compared vehicle (V) versus fruticulin A treated groups and # compared the control group before (basal) and after TNF or L-DOPA treatment. ^#^ *P* < 0.001, ^*^ *P* < 0.05, difference in comparison with basal threshold levels or the vehicle-treated group. Differences between groups were analyzed by analysis of variance (one-way ANOVA) followed by the Newman-Keuls test.

TNF injection decreased significantly the basal values of measure by electronic Von Frey mechanical stimulation. Local administration of** 1** inhibited the hyperalgesic effects of TNF, but not L-DOPA, significantly preventing the decrease of the threshold of sensitivity, being of 72 ± 7 at a dose of 1 *μ*g/paw, after 3 hours of TNF injection, with *P* < 0.05 (Figure 8).

## 4. Discussion

The chemistry of* Salvia* spp. is very diverse, being characterized by accumulation of essential oils, flavonoids, diterpenes, and triterpenes. These compound classes are responsible by anti-inflammatory and antinociceptive activities that have been reported for several* Salvia* species, such as* S. officinalis, S. plebeia, S. miltiorriza, S. leriifolia, S. hypoleuca, S. divinorum, S. splendens*, and* S. bicolor *[3, 15–27].

In this work, the ethanolic extract of* S. lachnostachys* leaves was able to inhibit some parameters of inflammation such as mechanical pain, leukocyte migration, oedema, and plasma leakage in experimental inflammatory models. These activities can be associated with the presence of oleanolic and ursolic acids, two triterpenes common in* Salvia*, which are well known as anti-inflammatory agents [3]. The diterpene fruticulin A also was identified as one major compound in the ethanolic extract of* S. lachnostachys*. Fruticulin A is a rare compound and was reported in only two other species of* Salvia, S. fruticulosa* [28] and* S. corrugata* [29]. Previous studies showed antibacterial [29, 30] and cancer chemopreventive activities of fruticulin A [31], but anti-inflammatory assays had not been performed with this compound. To test the contribution of fruticulin A to the activities observed in the extract, we tested the compound for anti-inflammatory and antihyperalgesic activities. The results showed that fruticulin A reduces the oedema, leukocyte migration, protein leakage, and hypernociception induced by carrageenan. Fruticulin A also showed an antihyperalgesic effect when administered locally, inhibiting both carrageenan and TNF induced hyperalgesia.

Carrageenan induces inflammatory pain by a mechanism of nociceptor sensibilization [12]. Oedema formation induced by carrageenan is described as a biphasic event: the first phase (0–2 hours) is mediated by histamine and serotonin followed by bradykinin, prostaglandins, and lysosome, while the late phase (2–6 hours) is related to the production of TNF, interleukins (IL-1*β* and IL-6), and nitric oxide [32]. Similarly, carrageenan-induced hypernociception also depends on mediators like bradykinin, TNF, prostaglandin, keratinocyte-derived chemokine (KC), and sympathetic amines [13, 14].

TNF is produced by mononuclear phagocytes and is responsible for immune responses and induction of other inflammatory cytokines [33]. Therefore, TNF may be involved in both oedema formation and hyperalgesia, which may explain the effects of fruticulin A on animal models performed. The other pathway of carrageenan-induced hyperalgesia involves the sympathetic amines, and L-DOPA injection activates this pathway, but fruticulin A did not interfere in this activation showing that this pathway is not important for fruticulin A antihyperalgesic effects. These results suggest that the main mechanism of fruticulin A antihyperalgesic properties could involve TNF pathway.

## 5. Conclusion

The present study showed the ethanolic extract from* S. lachnostachys* leaves (SLEE) to have anti-inflammatory and antihyperalgesic effects in oedema, pleurisy, and hyperalgesia induced by carrageenan models in mice. The observed activities can be related to presence of the triterpenes oleanolic and ursolic acids and of the diterpene fruticulin A. The last one showed significant anti-inflammatory and antihyperalgesic activities, and TNF seems to be involved in its mechanism of action. This study is the first report on the pharmacological properties of* S. lachnostachys* and fruticulin A.
